# Genetic Variation in *Neisseria meningitidis* Does Not Influence Disease Severity in Meningococcal Meningitis

**DOI:** 10.3389/fmed.2020.594769

**Published:** 2020-11-11

**Authors:** Philip H. C. Kremer, John A. Lees, Bart Ferwerda, Arie van de Ende, Matthijs C. Brouwer, Stephen D. Bentley, Diederik van de Beek

**Affiliations:** ^1^Department of Neurology, Amsterdam Neuroscience, Amsterdam University Medical Center, University of Amsterdam, Amsterdam, Netherlands; ^2^Parasites and Microbes, Wellcome Sanger Institute, Cambridge, United Kingdom; ^3^Department of Infectious Disease Epidemiology, Medical Research Council Centre for Global Infectious Disease Analysis, Imperial College London, London, United Kingdom; ^4^Department of Clinical Epidemiology, Biostatistics and Bioinformatics, Amsterdam University Medical Center, University of Amsterdam, Amsterdam, Netherlands; ^5^Department of Medical Microbiology and Infection Prevention, Amsterdam University Medical Center, University of Amsterdam, Amsterdam, Netherlands; ^6^The Netherlands Reference Laboratory for Bacterial Meningitis, Amsterdam, Netherlands

**Keywords:** severity, genome-wide association study, genome sequencing, *Neisseria meningitidis*, thrombocytes

## Abstract

*Neisseria meningitidis* causes sepsis and meningitis in humans. It has been suggested that pathogen genetic variation determines variance in disease severity. Here we report results of a genome-wide association study of 486 *N. meningitidis* genomes from meningococcal meningitis patients and their association with disease severity. Of 369 meningococcal meningitis patients for whom clinical data was available, 44 (12%) had unfavorable outcome and 24 (7%) died. To increase power, thrombocyte count was used as proxy marker for disease severity. Bacterial genetic variants were called as k-mers, SNPs, insertions and deletions and clusters of orthologous genes (COGs). Population-level meningococcal genetic variation did not explain variance in disease severity (unfavorable outcome or thrombocyte count) in this cohort (h^2^ = 0.0%; 95% confidence interval: 0.0–0.9). Genetic variants in the bacterial *uppS* gene represented the top signal associated with thrombocyte count (*p*-value = 9.96e-07) but this did not reach statistical significance. We did not find an association between previously published variants in *lpxL1, fHbp*, and *tps* genes and unfavorable outcome or thrombocyte count. A power analysis based on simulated phenotypes based on real genetic data from 880 *N. meningitidis* genomes showed that we would be able to detect a continuous phenotype with h^2^ > = 0.5 with the population size available in this study. This rules out a major contribution of pathogen genetic variation to disease severity in meningococcal meningitis, and shows that much larger sample sizes are required to find specific low-effect genetic variants modulating disease outcome in meningococcal meningitis.

## Introduction

*Neisseria meningitidis* is a commensal to the human nasopharynx (1). Rarely, it crosses the mucosal barrier to cause invasive meningococcal disease, which can manifest as bacteremia, fulminant septicemia and meningitis (2). Meningitis occurs in over 60% of patients with invasive meningococcal disease, dependent on the income level of a country and patient age, and is invariably preceded by bacteremia (2, 3). In fulminant meningococcal septicemia, patients present with severe septic shock and no clinical signs of meningitis. In cerebrospinal fluid, few meningococci are present and white cell count is low (4, 5). Contrary, patients with meningococcal meningitis have lower concentrations of meningococci and endotoxin in blood, but higher concentrations in cerebrospinal fluid (CSF) (5, 6). For meningococcal septicemia, mortality rate is around 12% (7). For meningitis, mortality rate is lower, around 3%; and 10% of patients have neurological sequelae after disease (8). Unfavorable outcome in patients with meningococcal meningitis is the result of neurological or systemic disease complications such as multi-organ failure complicating bacteremia, peripheral vasculopathy and peripheral ischemia (9). Lipopolysaccharide (LPS) is a major component of the meningococcal outer membrane. In the host, LPS is recognized by Toll-like receptor 4 (TLR4). Activation of TLR4 results in induction of the innate immune system and activation of the coagulation system through upregulation of tissue-factor (10). Excessive activation of the coagulation system can result in disseminated intravascular coagulation (DIC). This leads to low blood thrombocyte counts through consumption. Low blood thrombocyte counts are associated with severe meningococcal disease and unfavorable outcome (11).

In meningococcal meningitis, host genes involved in inflammation and coagulation have been suggested to be involved in severity of disease (12). Polymorphisms in SERPINE1, a gene in the fibrinolysis pathway, and interleukin-1B (IL1B) and IL1RN, mediating cytokine production, were associated with mortality in a meta-analysis (12). Multiple meningococcal virulence factors have been identified (13). For a minority of these, an association with disease severity in humans has been determined. Mutations in the bacterial gene *lpxL1*, resulting in penta-acetylated LPS vs. wild-type hexa-acetylated LPS, have been shown to lead to decreased systemic inflammation and reduced coagulation, resulting in higher blood thrombocyte counts and less unfavorable outcome in patients (14). Meningococcal factor H binding protein (FHbp) inhibits complement activation by binding to human factor H. The *fHbp* gene has high sequence diversity and a study of this variability revealed an association with disease severity and outcome, but not with blood thrombocyte counts (15). Finally, gene variants encoding a two-partner secretion system have been implicated in disease severity, through an unknown mechanism of action (16).

We performed a pathogen genome-wide association study in meningococcal isolates causing meningitis to determine the contribution of genetic variants in explaining disease severity.

## Methods

### Nationwide Clinical Cohort

Adults aged 16 years or older who had *N. meningitidis* meningitis were identified by positive cerebrospinal fluid (CSF) culture, and were listed in the database of The Netherlands Reference Laboratory for Bacterial Meningitis (NRLBM) between 1982 and 2003 and between 2006 and 2013. Between 1 October 1998 and 1 April 2002 (17) and between 1 March 2006 and 1 April 2012 (8) two prospective national cohort studies ran in which patients with bacterial meningitis were included and their clinical characteristics were collected through a case record form. Patients with hospital-acquired bacterial meningitis, neurosurgical procedures, or those within 1 month following neurosurgical procedure or neurotrauma were excluded. Patients with an altered immune status owing to splenectomy, diabetes mellitus, cancer, alcoholism, or the use of immunosuppressive drugs were considered immunocompromised, as were patients infected with human immunodeficiency virus. Neurological examination was performed at discharge, and outcome was scored according to the Glasgow Outcome Scale, with scores varying from 1 (death) to 5 (good recovery) (18). A favorable outcome was defined as a score of 5, and an unfavorable outcome was defined as a score of 1–4. Seizures were defined as a clinical diagnosis of an epileptic seizure at or during hospital admittance, with or without electroencephalographic (EEG) confirmation. Thrombocyte count (10^9^ per liter [L]), c-reactive protein (CRP; milligrams [mg] per L), cerebrospinal fluid (CSF) leucocytes (per microliter [uL]), CSF protein (grams [g] per L) and CSF glucose ratio were determined at admittance by the laboratory of the admitting hospital as part of routine clinical care. The NRLBM collects meningococcal isolates from cerebrospinal fluid, blood, and skin biopsy from clinical microbiology laboratories throughout the Netherlands. During the inclusion periods, notification for this disease was mandatory.

### Ethical Approval

Written informed consent was obtained from all patients or their legally authorized representatives. The studies were approved by the Medical Ethics Committee of the Amsterdam UMC, Amsterdam, The Netherlands (approval number: NL43784.018.13).

### Bacterial Whole Genome Sequencing

DNA from *N. meningitidis* strains was extracted using the Maxwell® RSC Cultured Cells DNA kit according to the manufacturer's protocol (Promega, Madison, WI, USA). Sequencing was performed using multiplexed libraries on the Illumina HiSeq platform to produce paired-end reads of 100 nucleotides in length (Illumina, San Diego, CA, USA). Quality control involved analysis of contamination, number and length of contigs, GC content and N50 parameter. Sequences for which one or more of these quality control parameters deviated by more than 3 standard deviations from the mean, were excluded. Sequences of the bacterial samples were assembled using a standard assembly pipeline (19). The median number of contigs was 85 (range 54–133), median GC content 53.83% (range 53.43–54.00%), average genome length 2,160,459 (range 2 066 672–2 389 876), and median coverage 204-fold (interquartile range 193–216). Serogroups and sequence types were determined from the whole genome sequence by in-house scripts. Clonal complexes were determined from sequence types.

### Data Availability

Fastq sequences of bacterial isolates were deposited in the European Nucleotide Archive (ENA, accession numbers in Supplementary Table 1).

### Pan-Genome Generation and Phylogenetic Tree

Genome sequences were annotated with PROKKA, version 1.11 (20). Roary (version 3.5.0) with default parameters was used to extract clusters of orthologous genes, and create a core gene alignment at a sequence identity threshold of 95% (21). A maximum likelihood phylogeny of single-nucleotide polymorphisms (SNPs) in the core genome of all sequenced isolates was produced with iqtree (version 1.6.5, including fast stochastic tree search algorithm) assuming a general time reversible model of nucleotide substitution with a discrete γ-distributed rate heterogeneity and the allowance of invariable sites (22).

### Genetic Variants and Association Analysis

Sequence variation was determined in multiple ways. To obtain a reference free compilation of genetic variation, encompassing single and multiple base pair variants, we determined non-redundant k-mers from assembled sequences by counting nodes on compacted De Bruijn graphs with Unitig-counter (version 1.0.5, minimum k-mer length of 13) (23). SNPs and rare variants [deleterious variants with a minor allele frequency (MAF) <0.01] were called separately with Snippy (version 4.4.0, default parameters) from whole-genome sequence reads. Genetic variation was called against the *N. meningitidis* MC58 reference strain (24). Clusters of orthologous genes (COGs) were determined with Roary, with a sequence identity threshold of 95% (21). The association analysis for k-mers and SNPs was run as a linear mixed model in Pyseer (version 1.1.1), with a minor allele frequency of 0.05 (25). To correct for population structure, a similarity matrix, generated with Pyseer, was included. K-mers were mapped to the MC58 reference strain with bowtie-2 (version 2.2.3, with equal quality values and length of seed substrings 7 nucleotides). A *p*-value of 0.05 corrected for the number of independent tests defined as the number of unique k-mer patterns was selected as a threshold for association of the phenotype with k-mers. For SNPs, rare variants, and for COGs, a *p*-value of 0.05 divided by the number of statistical tests performed at the pre-specified minor allele frequency was selected as a threshold for association. Rare variants were considered as a burden test in which they were grouped per gene. Manhattan plots were generated in R, version 3.5.1, with the package ggplot2 (version 3.1.0). Quantile-quantile plots for SNP and k-mer data were generated in R using the qqman (version 0.1.7) package. The association with clinical characteristics was determined by Fisher's exact test for categorical data, Wald test for continuous data and binary logistic regression for multivariate analysis in R.

For the candidate gene analyses, results of the k-mer and SNP associations with phenotype in the genome-wide association analysis were extracted at the *lpxL1* and *fHbp* gene locations in the MC58 reference genome. The number of independent tests was defined as the number of genetic variants in the respective locus and the threshold for statistical significance was set accordingly. Presence or absence of the *tps* variants were determined as COGs. Association analyses were performed in Pyseer (version 1.1.1) as linear mixed models, accounting for population stratification as described before. SNP-based heritability analyses were performed with a linear mixed model in Pyseer (version 1.1.1) using the genomic relatedness matrix as random effects (25). A confidence interval around these values was determined with ALBI (default parameters) based on the eigenvalue decomposed distances in the kinship matrix (26).

### GWAS Simulation

The power this dataset had to find genetic variants was quantified by running genome-wide association studies with simulated phenotypes at heritability values of 0.1, 0.2, 0.3, 0.4, and 0.5 in dataset of 880 whole genome sequences from publicly available *N. meningitidis* genomes (27). Simulations of continuous phenotypes were generated using GCTA (version 1.93.2 beta), using ten simulation replicates and either 10 or 1,000 random causal SNPs with effect sizes randomly generated from a standard normal distribution). Plots were generated in R (version 4.0.0), with the package ggplot2 (version 3.1.0).

## Results

### Clinical Characteristics

Between 1998 and 2002, 258 of 696 (37%) and between 2006 and 2013, 150 of 1,604 (9%) patients with meningitis had *N. meningitidis* as the causative pathogen. Meningococci from 78 patients out of an unknown total were included with isolation dates before 1998. Clinical data, including disease outcome, was available for 369 of 486 patients of whom meningococcal isolates were available. The median age was 29 years (interquartile range 19–51) and 167 (49%) were female (Table 1). Forty four patients (12%) had an unfavorable outcome and 24 (7%) died. Of the 486 isolates, 354 (73%) were serogroup B and 107 (22%) serogroup C. The remaining 25 isolates (5%) were serogroup A, W, X, Y, or E (28). The genotyping alleles of the major antigens have been described elsewhere (28). Unfavorable outcome was the result of serogroup C [13 out of 69 (19%)] and ST-11 complex [15 out of 85 (18%)] infection more often compared to other serogroups and clonal complexes (Supplementary Table 2). To increase the power to identify genetic variations associated with disease severity we investigated whether blood thrombocyte count on admission was a predictor of unfavorable outcome. We found that unfavorable outcome was highly correlated with a low thrombocyte count in blood (Wald test, *p* < 0.001, *n* = 342; Supplementary Figure 1). While age was also associated with unfavorable outcome (Wald test, *p* < 0.001, *n* = 364), age was not associated with thrombocyte count (Kruskal-Wallis test, *p* = 0.778). Sex was not associated with clinical outcome (chi-square test, *p* = 0.140) or thrombocyte count (Kruskal-Wallis test, *p* = 0.519). Clinical presentation was associated with unfavorable outcome, as well as thrombocyte count, because it is part of the same pathway of the phenotype under study (disease severity).

**Table 1:** Baseline characteristics of patients.

**Clinical characteristic**	
**Inclusion period (N/N evaluated)**	
Before 1998	78/unknown
1998–2002	258/696
2006–2013	150/1,604
Unfavorable outcome (number, %)	44 (12%)
Age in years (median, IQR)	29 (19–51)
Female sex (number, %)	167 (49%)
Seizures (number, %)	6 (2%)
Immunocompromise (number, %)	27 (8%)
Symptom duration <24 h	162 (49%)
CRP (median mg L^−1^, IQR)	227 (156–315)
CSF leucocytes (median uL^−1^, IQR)	5,306 (1,523–13,271)
CSF protein (median g L^−1^, IQR)	4.2 (2.0–6.7)
CSF glucose ratio (median, IQR)	0.08 (0.01–0.32)
Dexamethasone treatment (number, %)	139 (41%)
**Clonal complex (number, %)**	
ST-41/44	128 (38%)
ST-32	65 (19%)
ST-11	65 (19%)
ST-269	23 (7%)
ST-213	12 (4%)
Other	49 (14%)

*IQR, interquartile range; CRP, C-reactive protein; CSF, cerebrospinal fluid; mg, milligrams; mL, milliliter; g, grams; uL, microliter; ST, sequence type. Numbers might not add up to total number of patients because of missing data*.

### No Variants Surpass Threshold for Genome-Wide Significance in a Pathogen Genome-Wide Association Analysis

**Figure 1:**
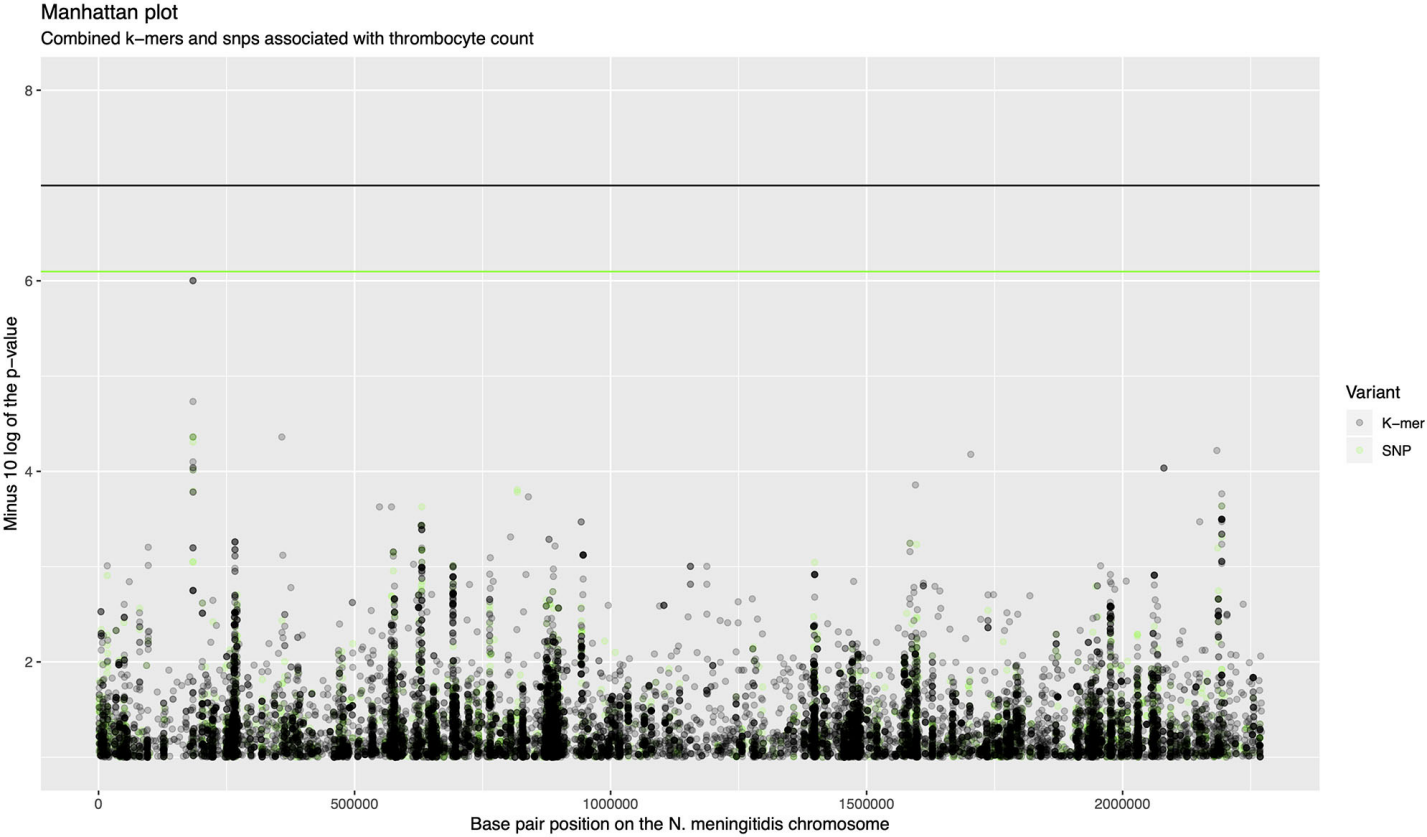
Manhattan plot of combined k-mers and SNPs associated with thrombocyte count. The x-axis shows the base pair position on the *N. meningitidis* chromosome. The y-axis shows the minus log 10 transformed *p*-value. K-mers are shown in black, SNPs are shown in green. The region around 200,000 base pairs is the top signal for association with thrombocyte count.

The association analysis was performed on 342 samples for which thrombocyte count as a continues variable was available. There were 611,389 unique k-mer patterns which varied in length from 13 to 46 nucleotides derived from bacterial sequences combined. After calling SNPs, there were 170,582 nucleotide variable positions (single nucleotide variants, insertions and deletions), of which 64,616 were present in between 5 and 95% of sequences. A *p*-value of 1.0 × 10^−7^ was selected as a threshold for association of the phenotype with k-mers and a *p*-value of 8.0 × 10^−7^ for association with SNPs. There were 1,445 rare variants called from the SNP data which were burdened in 260 genes. One thousand seven hundred and seventy five COGs were considered in association with the phenotype. None of the variants in the *N. meningitidis* genome surpassed the threshold for association after correction for multiple testing (Figure 1, Supplementary Figure 2). The top signal associated with thrombocyte count was a region at approximately 200,000 base pair positions in the *N. meningitidis* genome (*p* = 9.96e-07, Figure 1). This region held an isoprenyl trasferase (*uppS*) gene (Supplementary Figure 3).

### Isoprenyl Trasferase (uppS) Gene Variation Is Associated With High Thrombocyte Count

**Figure 2:**
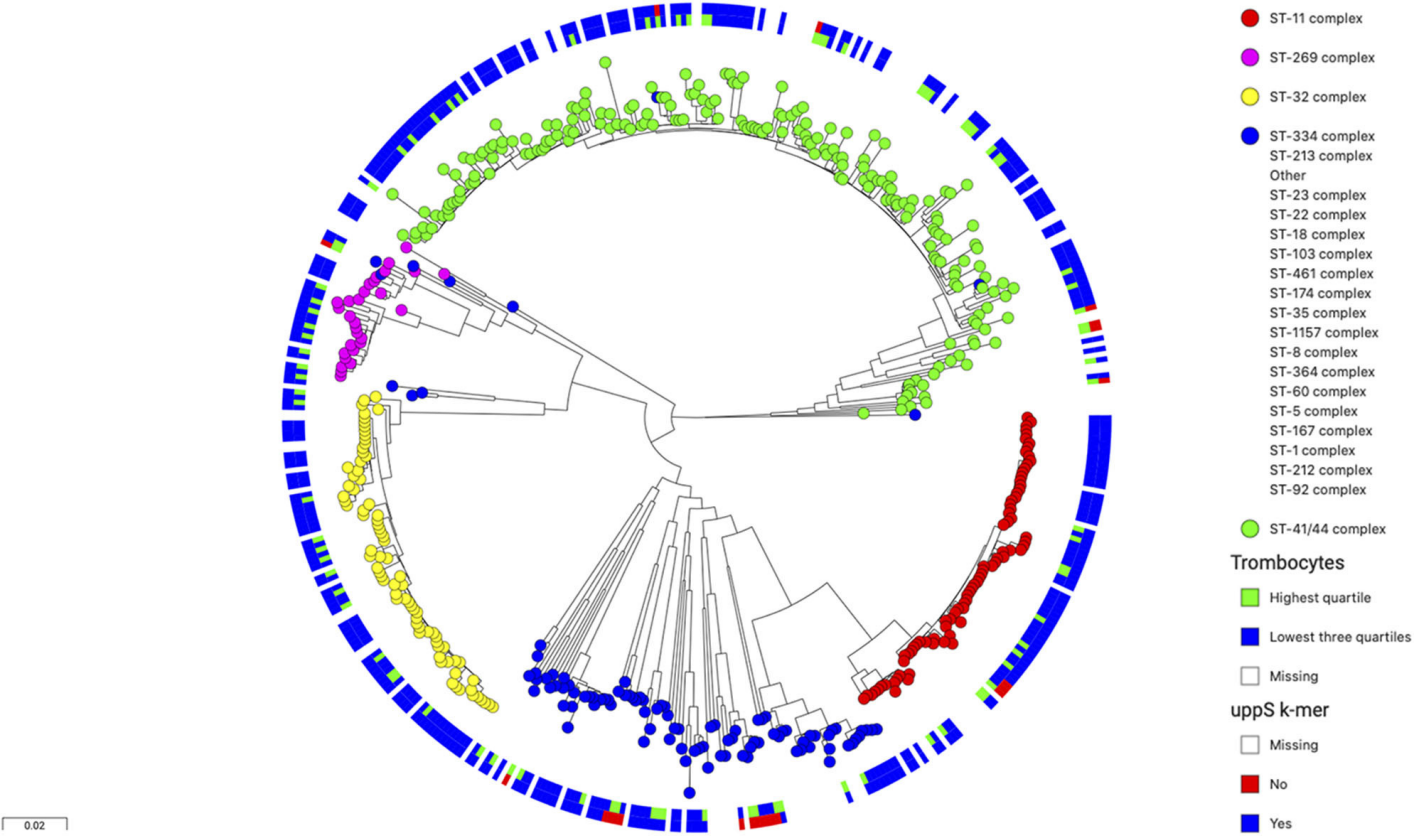
Phylogenetic tree of 486 N. meningitidis isolates colored by clonal complex. The inner circle shows isolates from patients with thrombocyte counts in blood (*n* = 342) in the lowest three quartiles in blue and the highest quartile in green. The outer circle shows isolates with wild-type *uppS* in blue and variant *uppS* in red. The variant *uppS* is found more often in isolates from patients with the highest thrombocyte quartile and is distributed evenly over the various clonal complexes in the tree.

The isoprenyl transferase gene is 746 nucleotides in length. The k-mer with the top signal in the *uppS* gene was 23 nucleotides long was annotated at position 146–168 nucleotides. The genetic variant of interest was absence of the k-mer sequence from the gene. Isolates from which the k-mer sequence was absent (which thus carried the variant of interest) had sequences which differed in one or two nucleotide positions (Table 2). These variant nucleotide positions were synonymous mutations. The variant (absence of the k-mer sequence) was rare and was found in 22 out of 342 isolates (6%). The variant was found more often in isolates from patients with a high blood thrombocyte count. In isolates from patients with a thrombocyte count in the highest quartile, the *uppS* variant was found in 14 of 86 isolates (16%), compared to 8 of 256 isolates (3%) from patients with thrombocyte counts in the lowest three quartiles. Isolates with the variant were detected as being evenly distributed over the phylogenetic tree (Figure 2).

**Table 2:** Nucleotide and amino acid sequence of the top associated k-mer sequence in *uppS* gene.

	**Associated sequence**	**Alternative sequence**
Nucleotide	AATTTCCTGCTCTGG CAGATGGC	AATTTCCTACTCTGG CAAATGGC AATTTCCTGCTTTGG CAGATGGC
Amino acid	NFLLWQM	NFLLWQM NFLLWQM

Patients infected with a *N. meningitidis* isolate containing the *uppS* variant had lower rate of unfavorable outcome compared to wild-type isolates [1 of 22 patients (5%), vs. 40 of 320 patients (13%), Fisher's exact test *p* = 0.231], were of female sex less often [7 of 22 (32%), vs. 160 of 320 (50%), Fisher's exact test *p* = 0.075], and had lower CRP in blood levels at admission [128 mg L^−1^, interquartile range (IQR) 22–182; vs. 230, IQR 164–320; Wald test *p* < 0.001], lower CSF leucocyte levels at admission (2,363 uL^−1^, IQR 1,579–9,759; vs. 5,461, IQR 1,527–13,375; Wald test *p* = 0.132), and lower CSF protein levels at admission (3.1 g L^−1^, IQR 1.36–5.1; vs. 4.38, IQR 2.22–6.82; Wald test *p* = 0.037) (Table 3). CRP in blood was associated with *uppS* variant independently of thrombocyte count in a multivariate analysis (*p* < 0.001).

**Table 3:** Clinical characteristics in patients infected with meningococci with *uppS* wild-type or variant.

**Clinical characteristic**	***uppS* wild-type (n = 320)**	***uppS* variant (*n* = 22)**	***p*-value**	**Multivariate analysis**
CRP (median mg L^−1^, IQR)	230 (164–320)	128 (22–182)	<0.001^&^	<0.001^%^
CSF protein (median g L^−1^, IQR)	4.3 (2.1–6.9)	3.1 (1.4–5.1)	0.037^&^	N.S.^%^

*N, number; CRP, C-reactive protein; IQR, interquartile range; CSF, cerebrospinal fluid; mg, milligrams; mL, milliliter; g, grams; N.S., not significant Numbers do not add up to total number of patients because of missing values*.

& *Wald test p-value*;

% *multivariate logistic regression corrected for thrombocyte count*.

### Genetic Variance Does Not Account for Variance in Disease Severity

To determine whether the lack of genome-wide significant results was more likely due to low statistical power or whether it represents a true biological feature, we calculated the proportion of variance in blood thrombocyte count that was attributable to genetics in the *N. meningitidis* genome by calculating h^2^. Heritability for this phenotype was zero, with an h^2^ of 0.0% (95% confidence interval: 0.0–0.9), based on SNPs mapped against a reference genome as well as pan-genome variation covered by k-mers. The wide confidence interval around the heritability value suggests low detection power for causative variants.

### Phenotype Simulations Rule Out Large Contributions of Pathogen Genome Variation

**Figure 3:**
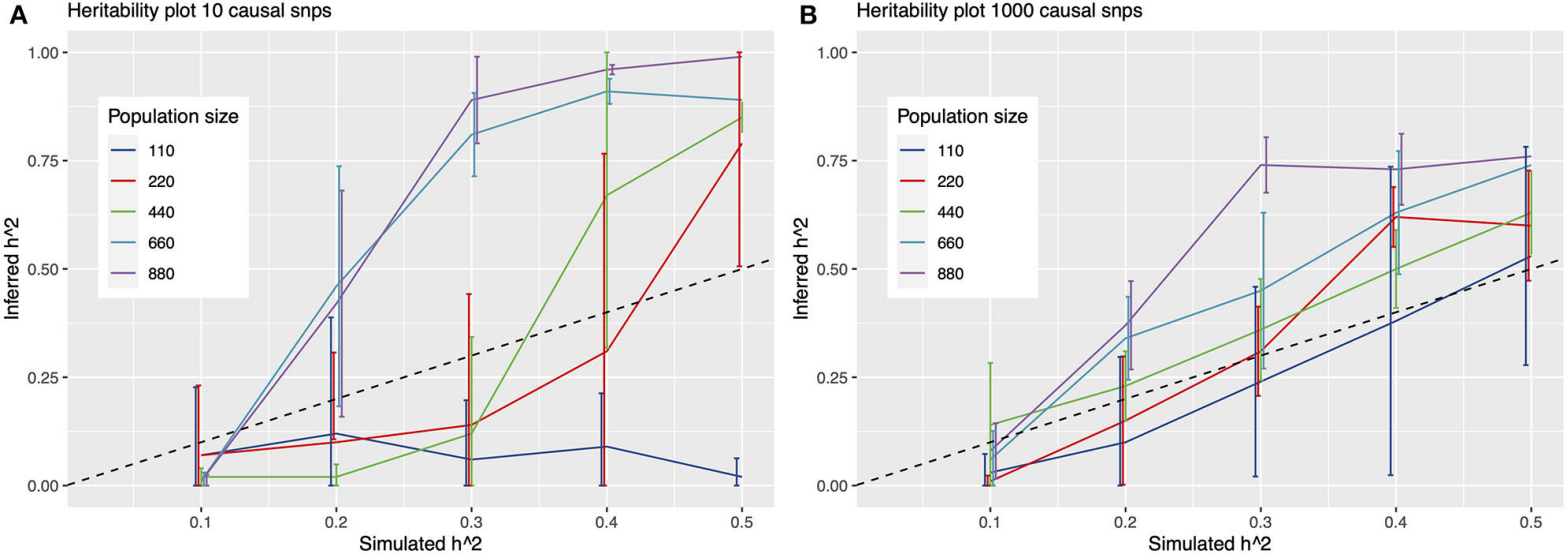
Heritability plots for a simulated GWAS with 10 **(A)** and 1,000 **(B)** causal SNPs. On the x-axis is the simulated heritability (h^2^) value. On the y-axis the inferred h^2^. Colored lines represent different population sizes. The dashed line represents a perfect fit, x = y. Detection of causal variants is limited for continuous traits with heritability values around 0.25 or lower at the sample size in this study (342 genomes), irrespective of assuming 10 or 1,000 causal variants under a polygenic model.

To further quantify this, we simulated phenotypes, and ran multiple GWAS on these simulations to determine detection power at fictitious heritability values in a cohort of 880 *N. meningococci* genomes. From these results, it emerges that detection of causal variants is limited for continuous traits with heritability values around 0.25 or lower at the current sample size, irrespective of the number of causal variants assumed under a polygenic model (Figure 3). For a binary trait, detection is more or less precluded at a sample size of 880 samples, even at simulated heritability values of 0.5, resulting in a mean inferred h^2^ of 0.00 [standard deviation (sd) 0.007] for a model of 1,000 causal variants and an inferred h^2^ of 0.01 (sd 0.025) for a model of 10 causal variants.

### No Replication of lpxL1, fHbp, and tps Gene Mutations on Outcome and Thrombocyte Count

In our cohort, we determined the association between published variants in *lpxL1, fHbp*, and *tps* genes and thrombocyte count and unfavorable outcome (14–16). In the *lpxL1* gene, we detected 2 SNPs, 5 insertion/deletions and 46 k-mer variants at sites or spanning sites described to inactivate the *lpxL1* gene in the previous publication (14). We did not observe an association between these variants with blood thrombocyte count or unfavorable outcome after correcting for multiple testing. In the *fHbp* gene, at the 184 amino acid position, we observed only lysine to arginine substitutions or deletions (15). We detected 24 SNPs, 23 insertions/deletions and 376 k-mer variants at other sites in the *fHbp* gene. The lysine to arginine variants were not associated with blood thrombocyte count or unfavorable outcome in a model accounting for bacterial population stratification. Presence of *tps2* and *tps3* gene was not associated with less severe disease or thrombocyte count in this cohort (16).

## Discussion

In this pathogen genome-wide association analysis, we could not detect genetic loci in the meningococcal genome predictive of blood thrombocyte count as a proxy for disease severity. Our results show that much larger sample sizes are needed to detect genetic variants for disease severity, a trait with low heritability. Together with the failed replication of previously published gene signals leads us to conclude that the contribution of genetic variants in the meningococcal genome to disease severity is limited. Presumably, environmental factors, host genetics or interacting factors play a much larger role in determining disease course.

We found a nucleotide sequence in the *uppS* gene as the top signal associated with thrombocyte count. This isoprenyl transferase gene is involved in biosynthesis of terpenoids. While this sequence did not surpass the genome-wide threshold for significance, patients infected with meningococci absent for this sequence (variant) in the *uppS* gene had lower CRP levels in blood, independent of thrombocyte count, in conjunction with higher blood thrombocyte counts. These measures can be indicators of less severe disease. The variant itself is not causal, as the translated alternative amino acid sequence is identical. Further studies are needed to determine whether the variant marks a sequence in *uppS* or in another gene and whether it determines virulence.

We were unable to confirm mutations in the *lpxL1* gene associated with higher thrombocyte count in a previously published paper (14), both in a genome-wide approach and in a candidate gene approach. The study by Fransen et al. included samples from the same nationwide cohort from 1998 to 2002. The current study is larger and included the majority of samples from 2006 to 2013. By calling genetic variants as k-mers, SNPs, rare-variants and COGs we explored a broader range of genetic variability, increasing the multiple testing burden. Furthermore, in the study by Fransen et al., patients infected with meningococci having any *lpxL1* variant resulting in an inactive gene were compared with the group of patients with meningocococci with the active *lpxL1* gene. In contrast, in the present study, an association between thrombocyte count and each individual *lpxL1* variant was explored. We could not replicate earlier studies showing associations between *fHbp* and *tps* and outcome (15, 16). Changes in clonal complex distribution among the meningococcal isolates in the present study population compared to that used in the earlier studies may explain replication failure of those results.

The epidemiology of *N. meningitidis* meningitis in the Netherlands has previously been described (29). For serogroup B, a hyperendemic period started in 1982, peaked in 1993 and incidence subsequently decreased until the last observation in 2012. For serogroup C disease, vaccination was introduced in 2001 in response to an epidemic, which resulted in a major decrease in incidence (29). The samples in this study encompass these periods and represent a national and longitudinal meningococcal population. Because we correct for phylogenetic clusters in the genome-wide association analysis, bacterial lineages do not confound the results.

By simulating phenotypes and running a pathogen GWAS using a larger set of 880 unphenotyped genomes, we were able to provide approximations of the sample sizes required to detect traits depending on the level of heritability, and provide an upper bound on the heritability detectable in our set of 342 phenotyped isolates. We show that much larger sample sizes are needed to detect genetic variants for disease severity, a trait with low heritability, and that these data can be of added value in future genetic studies of *N. meningitidis*, for example a meta-analysis to increase study power.

Similar to what is found in this study, pathogen genetics did not explain the variance in disease severity in pneumococcal meningitis and no loci were detected to be associated with disease severity in a pathogen genome-wide association study in pneumococcal meningitis (30). In contrast, genetic variance in the pathogen does play a major role in susceptibility to pneumococcal meningitis (30). One explanation for this is that contrary to nasopharyngeal carriage, invasive disease is an evolutionary dead-end for bacteria, in which mutations resulting in excessive disease severity are not selected for. Host genetic variance did explain a proportion of the variance in pneumococcal disease severity (30). For meningococcal meningitis, loci within the complement factor H (CFH) gene and CFH-related protein 3 (CFHR3) in the host were associated with susceptibility to disease (31). We were unable to investigate host genetics as a contribution to meningococcal disease severity in this study because of small sample size.

The study has several limitations. First, the sample size, although considerable for meningococcal meningitis, is low for a pathogen genome-wide association analysis. Second, there was no validation cohort available. Third, meningococci have various mechanisms for genetic variation, resulting in decreased correlation between the genetic sequence and protein expression or structure and function. Major mechanisms for genetic variation in meningococci are phase variation (reversible switching of gene expression), transformation and homologous recombination (13), restriction-modification and epigenetics (32). These mechanisms limit the genetic detection of disease modifying loci.

Whole genome sequencing together with detailed clinical metadata enabled us to quantify the contribution of meningococcal genetic variants to meningococcal meningitis disease severity. For future studies, much larger datasets would be required. To obtain these, we recommend international collaborations resulting in datasets combining microbial genomics and clinical patient data.

## Data Availability Statement

The datasets generated for this study can be found in online repositories. The names of the repository/repositories and accession number(s) can be found in the article/Supplementary Material.

## Ethics Statement

The studies involving human participants were reviewed and approved by Medical Ethics Committee of the Amsterdam UMC, Amsterdam (approval number: NL43784.018.13). The patients/participants provided their written informed consent to participate in this study.

## Author Contributions

DB, SB, AE, and MB designed the study and collected the data. PK, JL, and BF performed the experiments. PK, JL, and BF analyzed the data. PK, JL, BF, MB, AE, SB, and DB interpreted the data. PK wrote the manuscript draft. JL, BF, MB, AE, SB, and DB provided comments on the manuscript. All authors contributed to the article and approved the submitted version.

## Conflict of Interest

The authors declare that the research was conducted in the absence of any commercial or financial relationships that could be construed as a potential conflict of interest.
