# Supplementary material for: Genetic Variation in Neisseria meningitidis Does Not Influence Disease Severity in Meningococcal Meningitis
Source: Front Med (Lausanne). 2020 Nov 11;7:594769. doi: 10.3389/fmed.2020.594769 (PMC7686797; doi:10.3389/fmed.2020.594769)
Supplement: Supplementary file 1 [file Data_Sheet_1.PDF]

Figure 1

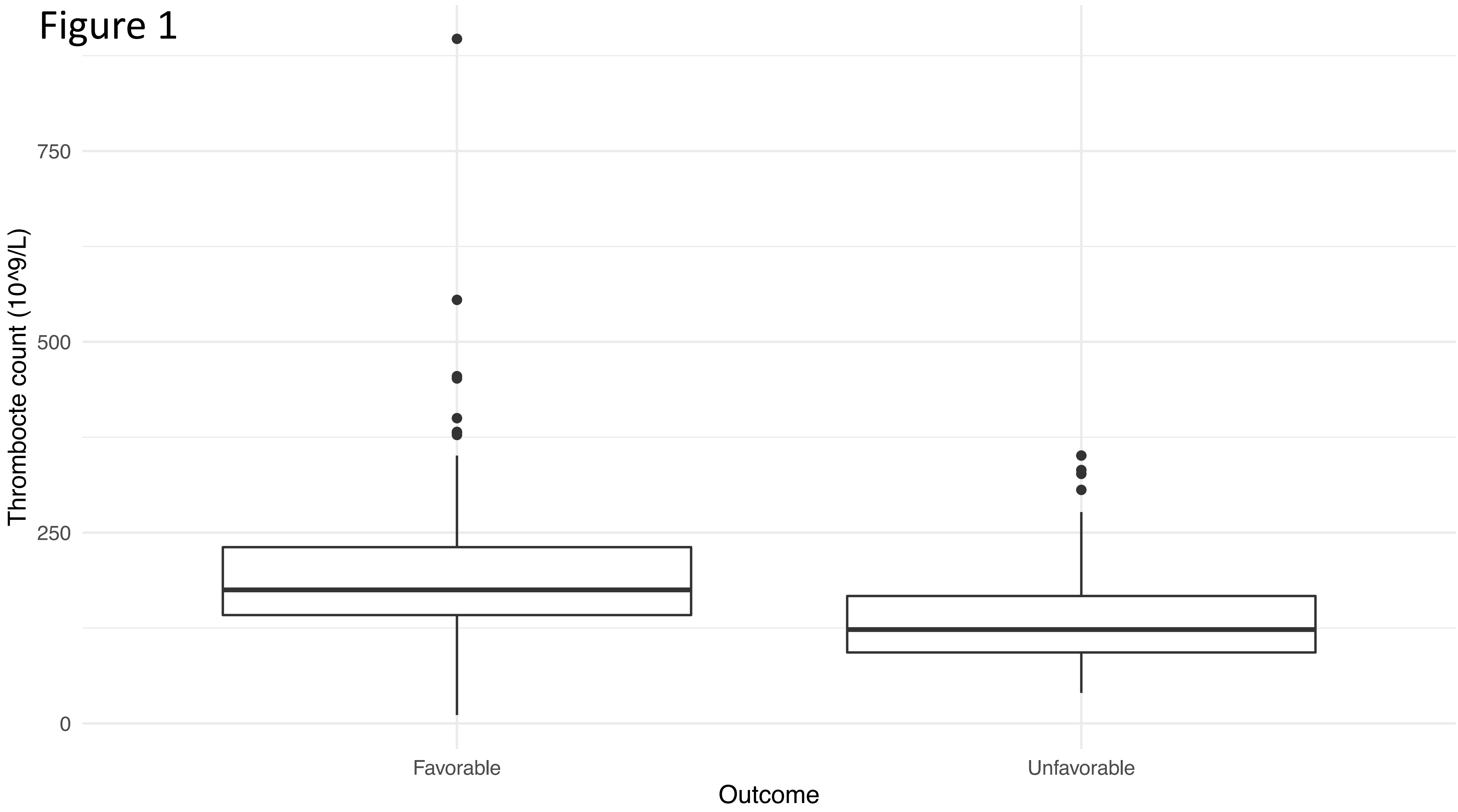

Thrombocte count (10^9/L)
750
500
250
0
Favorable
Unfavorable
Outcome

Figure 2

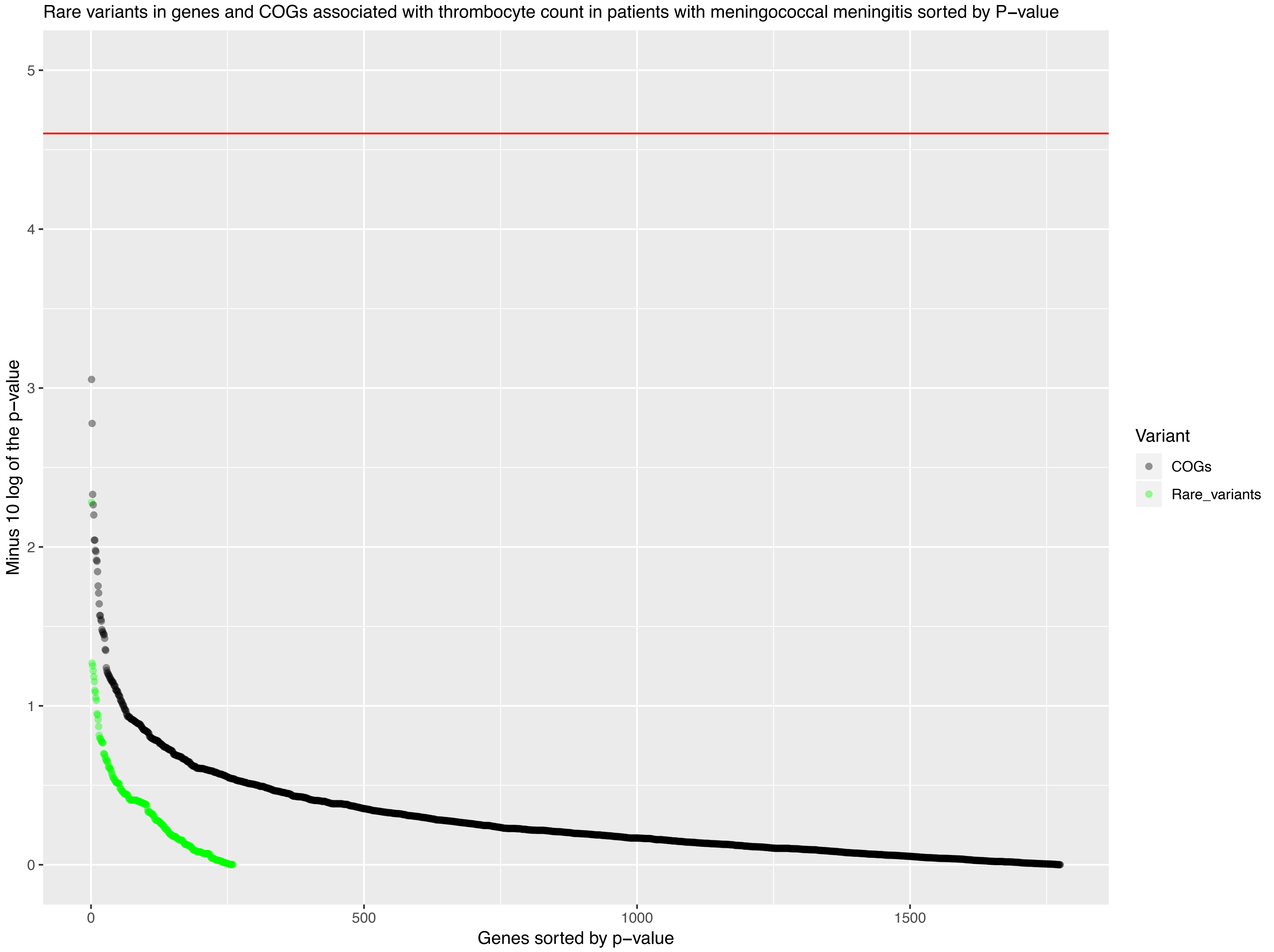
Rare variants in genes and COGs associated with thrombocyte count in patients with meningococcal meningitis sorted by P−value
Minus 10 log of the p−value
Genes sorted by p−value
0
1
2
3
4
5
0
500
1000
1500
Variant
COGs
Rare_variants

Figure 3

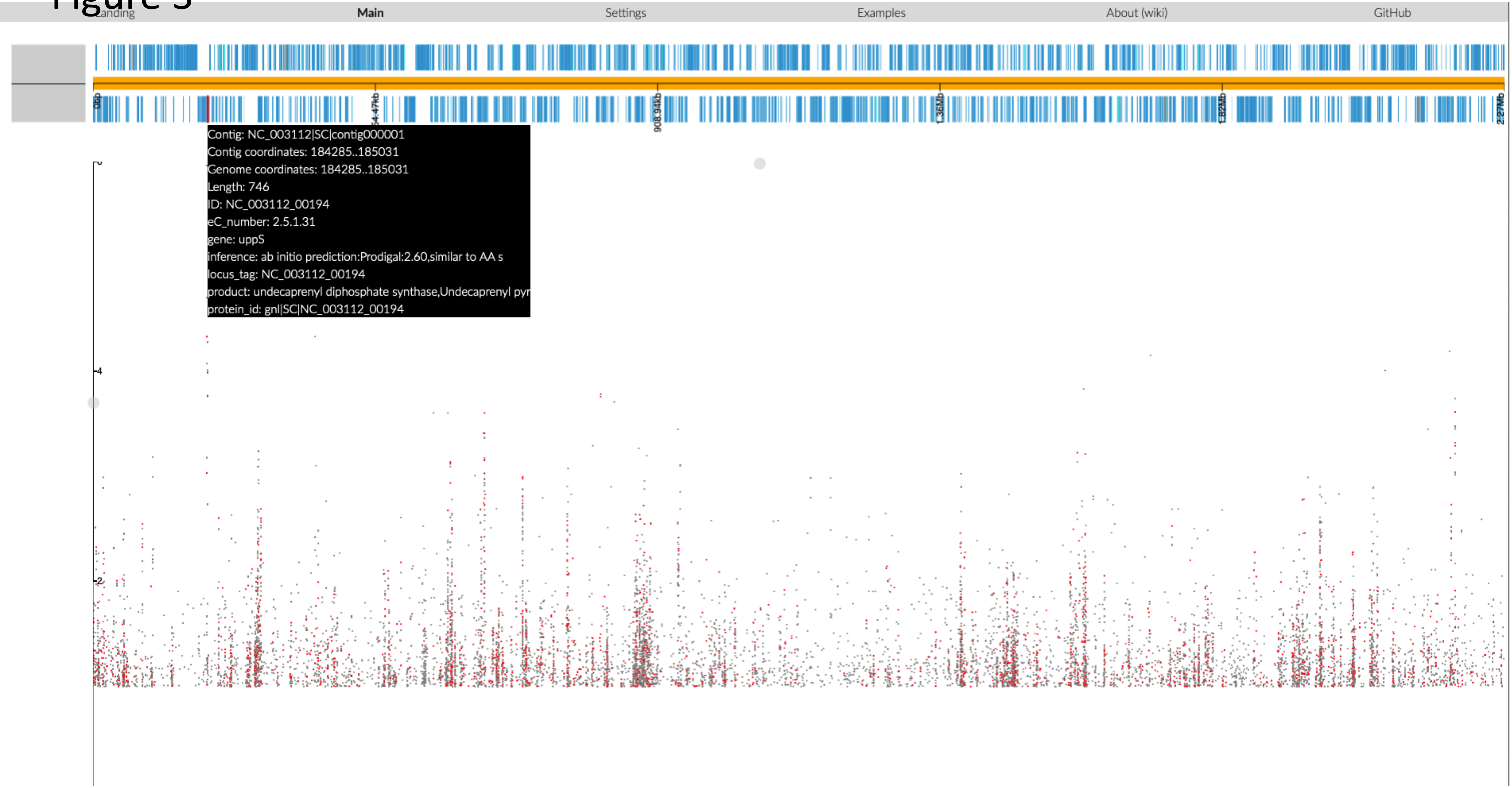

Landing
Main
Settings
Examples
About (wiki)
GitHub
0bp
454.47kb
908.94kb
1.36Mb
1.82Mb
2.27Mb
Contig: NC_003112|SC|contig000001
Contig coordinates: 184285..185031
Genome coordinates: 184285..185031
Length: 746
ID: NC_003112_00194
eC_number: 2.5.1.31
gene: uppS
inference: ab initio prediction:Prodigal:2.60,similar to AA s
locus_tag: NC_003112_00194
product: undecaprenyl diphosphate synthase,Undecaprenyl pyr
protein_id: gnl|SC|NC_003112_00194
4
2

Figure 4

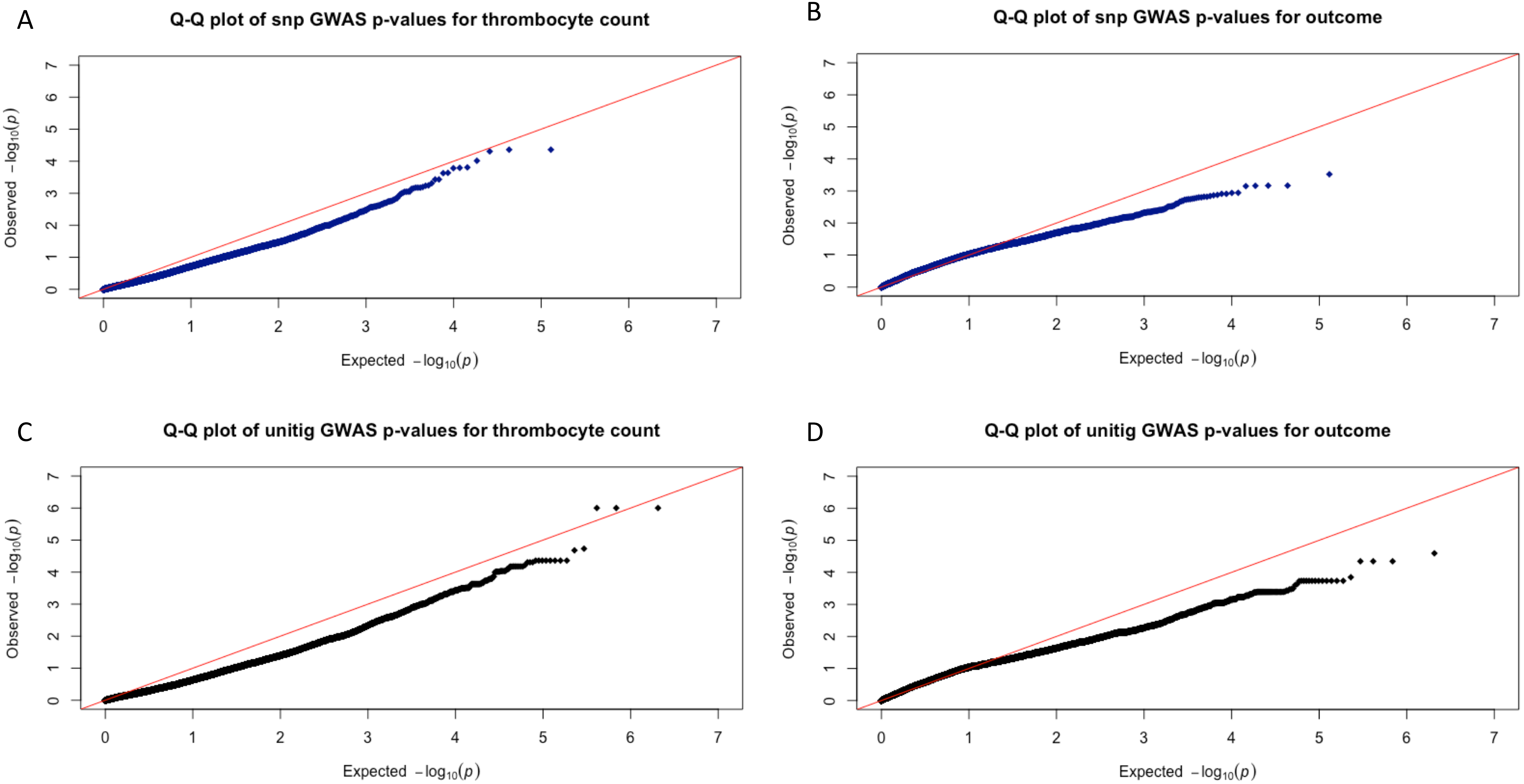
A
Q-Q plot of snp GWAS p-values for thrombocyte count
Observed $-\log_{10}(p)$
Expected $-\log_{10}(p)$
B
Q-Q plot of snp GWAS p-values for outcome
Observed $-\log_{10}(p)$
Expected $-\log_{10}(p)$
C
Q-Q plot of unitig GWAS p-values for thrombocyte count
Observed $-\log_{10}(p)$
Expected $-\log_{10}(p)$
D
Q-Q plot of unitig GWAS p-values for outcome
Observed $-\log_{10}(p)$
Expected $-\log_{10}(p)$
