# Supplementary material for: Genetic Variation in Neisseria meningitidis Does Not Influence Disease Severity in Meningococcal Meningitis
Source: Front Med (Lausanne). 2020 Nov 11;7:594769. doi: 10.3389/fmed.2020.594769 (PMC7686797; doi:10.3389/fmed.2020.594769)
Supplement: Supplementary file 2 [file Table_1.pdf]

Sample Acc	Lane Name	Lane Acc	Public Name	Study ID	Study Accession
ERS514568	14324_4#1	ERR708610	2071283	2842	ERP004245
ERS514569	14324_4#2	ERR708611	2071328	2842	ERP004245
ERS514570	14324_4#3	ERR708612	2071344	2842	ERP004245
ERS514571	14324_4#4	ERR708613	2071345	2842	ERP004245
ERS514572	14324_4#5	ERR708614	2071354	2842	ERP004245
ERS514573	14324_4#6	ERR708615	2071442	2842	ERP004245
ERS514574	14324_4#7	ERR708616	2071538	2842	ERP004245
ERS514575	14324_4#8	ERR708617	2071749	2842	ERP004245
ERS514576	14324_4#9	ERR708618	2071759	2842	ERP004245
ERS514577	14324_4#10	ERR708619	2071794	2842	ERP004245
ERS514578	14324_4#11	ERR708620	2071864	2842	ERP004245
ERS514579	14324_4#12	ERR708621	2071909	2842	ERP004245
ERS514580	14324_4#13	ERR708622	2080098	2842	ERP004245
ERS514581	14324_4#14	ERR708623	2080184	2842	ERP004245
ERS514582	14324_4#15	ERR708624	2080418	2842	ERP004245
ERS514583	14324_4#16	ERR708625	2080520	2842	ERP004245
ERS514584	14324_4#17	ERR708626	2080543	2842	ERP004245
ERS514585	14324_4#18	ERR708627	2080556	2842	ERP004245
ERS514586	14324_4#19	ERR708628	2080584	2842	ERP004245
ERS514587	14324_4#20	ERR708629	2080856	2842	ERP004245
ERS514588	14324_4#21	ERR708630	2081063	2842	ERP004245
ERS514589	14324_4#22	ERR708631	2081105	2842	ERP004245
ERS514590	14324_4#23	ERR708632	2081595	2842	ERP004245
ERS514591	14324_4#24	ERR708633	2081653	2842	ERP004245
ERS514592	14324_4#25	ERR708634	2081656	2842	ERP004245
ERS514593	14324_4#26	ERR708635	2081977	2842	ERP004245
ERS514594	14324_4#27	ERR708636	2082123	2842	ERP004245
ERS514595	14324_4#28	ERR708637	2082183	2842	ERP004245
ERS514596	14324_4#29	ERR708638	2082258	2842	ERP004245
ERS514597	14324_4#30	ERR708639	2090237	2842	ERP004245
ERS514598	14324_4#31	ERR708640	2090353	2842	ERP004245
ERS514599	14324_4#32	ERR708641	2090516	2842	ERP004245
ERS514600	14324_4#33	ERR708642	2090911	2842	ERP004245
ERS514601	14324_4#34	ERR708643	2091136	2842	ERP004245
ERS514602	14324_4#35	ERR708644	2091153	2842	ERP004245
ERS514603	14324_4#36	ERR708645	2091198	2842	ERP004245
ERS514604	14324_4#37	ERR708646	2091661	2842	ERP004245
ERS514605	14324_4#38	ERR708647	2091724	2842	ERP004245
ERS514606	14324_4#39	ERR708648	2092127	2842	ERP004245
ERS514607	14324_4#40	ERR708649	2093373	2842	ERP004245
ERS514608	14324_4#41	ERR708650	2094485	2842	ERP004245
ERS514609	14324_4#42	ERR708651	2100125	2842	ERP004245
ERS514610	14324_4#43	ERR708652	2100157	2842	ERP004245
ERS514611	14324_4#44	ERR708653	2100282	2842	ERP004245
ERS514612	14324_4#45	ERR708654	2100381	2842	ERP004245

ERS514613	14324_4#46	ERR708655	2100528	2842	ERP004245
ERS514614	14324_4#47	ERR708656	2102065	2842	ERP004245
ERS514615	14324_4#48	ERR708657	2102083	2842	ERP004245
ERS514616	14324_4#49	ERR708658	2103113	2842	ERP004245
ERS514617	14324_4#50	ERR708659	2103119	2842	ERP004245
ERS514619	14324_4#51	ERR708660	2103251	2842	ERP004245
ERS514621	14324_4#52	ERR708661	2103437	2842	ERP004245
ERS514623	14324_4#53	ERR708662	2103596	2842	ERP004245
ERS514625	14324_4#54	ERR708663	2103742	2842	ERP004245
ERS514627	14324_4#55	ERR708664	2103747	2842	ERP004245
ERS514628	14324_4#56	ERR708665	2104325	2842	ERP004245
ERS514631	14324_4#57	ERR708666	2104366	2842	ERP004245
ERS514632	14324_4#58	ERR708667	2110092	2842	ERP004245
ERS514634	14324_4#59	ERR708668	2110188	2842	ERP004245
ERS514637	14324_4#60	ERR708669	2110538	2842	ERP004245
ERS514640	14324_4#61	ERR708670	2110673	2842	ERP004245
ERS514642	14324_4#62	ERR708671	2110842	2842	ERP004245
ERS514645	14324_4#63	ERR708672	2110915	2842	ERP004245
ERS514648	14324_4#64	ERR708673	2110916	2842	ERP004245
ERS514651	14324_4#65	ERR708674	2110947	2842	ERP004245
ERS514653	14324_4#66	ERR708675	2111171	2842	ERP004245
ERS514655	14324_4#67	ERR708676	2111581	2842	ERP004245
ERS514658	14324_4#68	ERR708677	2111622	2842	ERP004245
ERS514661	14324_4#69	ERR708678	2120056	2842	ERP004245
ERS514664	14324_4#70	ERR708679	2120194	2842	ERP004245
ERS514667	14324_4#71	ERR708680	2120265	2842	ERP004245
ERS514669	14324_4#72	ERR708681	2120394	2842	ERP004245
ERS514671	14324_4#73	ERR708682	2120484	2842	ERP004245
ERS514674	14324_4#74	ERR708683	2120492	2842	ERP004245
ERS514677	14324_4#75	ERR708684	2120610	2842	ERP004245
ERS514679	14324_4#76	ERR708685	2120785	2842	ERP004245
ERS514681	14324_4#77	ERR708686	2120864	2842	ERP004245
ERS514684	14324_4#78	ERR708687	2120910	2842	ERP004245
ERS514686	14324_4#79	ERR708688	2120945	2842	ERP004245
ERS514689	14324_4#80	ERR708689	2121404	2842	ERP004245
ERS514692	14324_4#81	ERR708690	2121460	2842	ERP004245
ERS514694	14324_4#82	ERR708691	2121477	2842	ERP004245
ERS514696	14324_4#83	ERR708692	2121528	2842	ERP004245
ERS514699	14324_4#84	ERR708693	2130047	2842	ERP004245
ERS514702	14324_4#85	ERR708694	2130367	2842	ERP004245
ERS514705	14324_4#86	ERR708695	2130695	2842	ERP004245
ERS514707	14324_4#87	ERR708696	2130749	2842	ERP004245
ERS514853	14324_5#1	ERR708705	851200 I	2842	ERP004245
ERS514855	14324_5#3	ERR708707	860054 I	2842	ERP004245
ERS514857	14324_5#5	ERR708709	860394 I	2842	ERP004245
ERS514859	14324_5#7	ERR708711	860524 I	2842	ERP004245

ERS514861	14324_5#9	ERR708713	861538 I		2842	ERP004245
ERS514863	14324_5#11	ERR708715	871080 I		2842	ERP004245
ERS514865	14324_5#13	ERR708717	871091 I		2842	ERP004245
ERS514867	14324_5#15	ERR708719	871912 I		2842	ERP004245
ERS514869	14324_5#17	ERR708721	880416 I		2842	ERP004245
ERS514872	14324_5#20	ERR708724	880417 I		2842	ERP004245
ERS514874	14324_5#22	ERR708726	880470 I		2842	ERP004245
ERS514876	14324_5#24	ERR708728	881965 I		2842	ERP004245
ERS514879	14324_5#27	ERR708731	882011 I		2842	ERP004245
ERS514881	14324_5#29	ERR708733	890042 I		2842	ERP004245
ERS514884	14324_5#32	ERR708736	890104 I		2842	ERP004245
ERS514886	14324_5#34	ERR708738	890681 I		2842	ERP004245
ERS514888	14324_5#36	ERR708740	892385 I		2842	ERP004245
ERS514890	14324_5#38	ERR708742	892559 I		2842	ERP004245
ERS514893	14324_5#41	ERR708745	900382 I		2842	ERP004245
ERS514896	14324_5#44	ERR708748	900761 I		2842	ERP004245
ERS514898	14324_5#46	ERR708750	900798 I		2842	ERP004245
ERS514900	14324_5#48	ERR708752	900932 I		2842	ERP004245
ERS514903	14324_5#51	ERR708755	901147 I		2842	ERP004245
ERS514906	14324_5#54	ERR708758	901168 I		2842	ERP004245
ERS514909	14324_5#57	ERR708761	901458 I		2842	ERP004245
ERS514912	14324_5#60	ERR708764	901523 I		2842	ERP004245
ERS514914	14324_5#62	ERR708766	901549 I		2842	ERP004245
ERS514916	14324_5#64	ERR708768	901833 I		2842	ERP004245
ERS514919	14324_5#67	ERR708771	902499 I		2842	ERP004245
ERS514922	14324_5#70	ERR708774	910039 I		2842	ERP004245
ERS514924	14324_5#72	ERR708776	910083 I		2842	ERP004245
ERS514926	14324_5#74	ERR708778	910221 I		2842	ERP004245
ERS514928	14324_5#76	ERR708780	910333 I		2842	ERP004245
ERS514931	14324_5#79	ERR708783	910763 I		2842	ERP004245
ERS514933	14324_5#81	ERR708785	911634 I		2842	ERP004245
ERS514935	14324_5#83	ERR708787	911665 I		2842	ERP004245
ERS514937	14324_5#85	ERR708789	911685 I		2842	ERP004245
ERS514939	14324_5#87	ERR708791	911687 I		2842	ERP004245
ERS514942	14324_5#90	ERR708794	920056 I		2842	ERP004245
ERS514944	14324_5#92	ERR708796	920293 I		2842	ERP004245
ERS514947	14324_5#95	ERR708799	920545 I		2842	ERP004245
ERS514284	14355_5#1	ERR715801		2000250	2842	ERP004245
ERS514287	14355_5#2	ERR715802		2000297	2842	ERP004245
ERS514290	14355_5#3	ERR715803		2000311	2842	ERP004245
ERS514294	14355_5#4	ERR715804		2000345	2842	ERP004245
ERS514297	14355_5#5	ERR715805		2000373	2842	ERP004245
ERS514300	14355_5#6	ERR715806		2000384	2842	ERP004245
ERS514304	14355_5#7	ERR715807		2000488	2842	ERP004245
ERS514307	14355_5#8	ERR715808		2000500	2842	ERP004245
ERS514310	14355_5#9	ERR715809		2000589	2842	ERP004245

ERS514313	14355_5#10	ERR715810	2000594	2842	ERP004245
ERS514316	14355_5#11	ERR715811	2000607	2842	ERP004245
ERS514318	14355_5#12	ERR715812	2000608	2842	ERP004245
ERS514321	14355_5#13	ERR715813	2000622	2842	ERP004245
ERS514325	14355_5#14	ERR715814	2000709	2842	ERP004245
ERS514328	14355_5#15	ERR715815	2000732	2842	ERP004245
ERS514331	14355_5#16	ERR715816	2000739	2842	ERP004245
ERS514336	14355_5#17	ERR715817	2000749	2842	ERP004245
ERS514338	14355_5#18	ERR715818	2000760	2842	ERP004245
ERS514341	14355_5#19	ERR715819	2000803	2842	ERP004245
ERS514345	14355_5#20	ERR715820	2000804	2842	ERP004245
ERS514348	14355_5#21	ERR715821	2000869	2842	ERP004245
ERS514351	14355_5#22	ERR715822	2000880	2842	ERP004245
ERS514354	14355_5#23	ERR715823	2000881	2842	ERP004245
ERS514357	14355_5#24	ERR715824	2000885	2842	ERP004245
ERS514360	14355_5#25	ERR715825	2000948	2842	ERP004245
ERS514364	14355_5#26	ERR715826	2000974	2842	ERP004245
ERS514367	14355_5#27	ERR715827	2000987	2842	ERP004245
ERS514371	14355_5#28	ERR715828	2001043	2842	ERP004245
ERS514374	14355_5#29	ERR715829	2001212	2842	ERP004245
ERS514377	14355_5#30	ERR715830	2001256	2842	ERP004245
ERS514381	14355_5#31	ERR715831	2001297	2842	ERP004245
ERS514384	14355_5#32	ERR715832	2001318	2842	ERP004245
ERS514387	14355_5#33	ERR715833	2001329	2842	ERP004245
ERS514391	14355_5#34	ERR715834	2001346	2842	ERP004245
ERS514394	14355_5#35	ERR715835	2001394	2842	ERP004245
ERS514397	14355_5#36	ERR715836	2001449	2842	ERP004245
ERS514400	14355_5#37	ERR715837	2001474	2842	ERP004245
ERS514404	14355_5#38	ERR715838	2001477	2842	ERP004245
ERS514407	14355_5#39	ERR715839	2001553	2842	ERP004245
ERS514410	14355_5#40	ERR715840	2001556	2842	ERP004245
ERS514413	14355_5#41	ERR715841	2001573	2842	ERP004245
ERS514416	14355_5#42	ERR715842	2001615	2842	ERP004245
ERS514419	14355_5#43	ERR715843	2001632	2842	ERP004245
ERS514422	14355_5#44	ERR715844	2001633	2842	ERP004245
ERS514425	14355_5#45	ERR715845	2001717	2842	ERP004245
ERS514429	14355_5#46	ERR715846	2001718	2842	ERP004245
ERS514433	14355_5#47	ERR715847	2001782	2842	ERP004245
ERS514436	14355_5#48	ERR715848	2001862	2842	ERP004245
ERS514440	14355_5#49	ERR715849	2001915	2842	ERP004245
ERS514443	14355_5#50	ERR715850	2001970	2842	ERP004245
ERS514447	14355_5#51	ERR715851	2002042	2842	ERP004245
ERS514452	14355_5#52	ERR715852	2002091	2842	ERP004245
ERS514455	14355_5#53	ERR715853	2002154	2842	ERP004245
ERS514459	14355_5#54	ERR715854	2002173	2842	ERP004245
ERS514461	14355_5#55	ERR715855	2010002	2842	ERP004245

ERS514464	14355_5#56	ERR715856	2010024	2842	ERP004245
ERS514468	14355_5#57	ERR715857	2010080	2842	ERP004245
ERS514472	14355_5#58	ERR715858	2010082	2842	ERP004245
ERS514476	14355_5#59	ERR715859	2010115	2842	ERP004245
ERS514480	14355_5#60	ERR715860	2010178	2842	ERP004245
ERS514483	14355_5#61	ERR715861	2010216	2842	ERP004245
ERS514486	14355_5#62	ERR715862	2010221	2842	ERP004245
ERS514490	14355_5#63	ERR715863	2010259	2842	ERP004245
ERS514493	14355_5#64	ERR715864	2010277	2842	ERP004245
ERS514495	14355_5#65	ERR715865	2010306	2842	ERP004245
ERS514498	14355_5#66	ERR715866	2010321	2842	ERP004245
ERS514503	14355_5#67	ERR715867	2010353	2842	ERP004245
ERS514506	14355_5#68	ERR715868	2010450	2842	ERP004245
ERS514509	14355_5#69	ERR715869	2010513	2842	ERP004245
ERS514512	14355_5#70	ERR715870	2010640	2842	ERP004245
ERS514515	14355_5#71	ERR715871	2010688	2842	ERP004245
ERS514518	14355_5#72	ERR715872	2010699	2842	ERP004245
ERS514522	14355_5#73	ERR715873	2010749	2842	ERP004245
ERS514525	14355_5#74	ERR715874	2010760	2842	ERP004245
ERS514528	14355_5#75	ERR715875	2010903	2842	ERP004245
ERS514530	14355_5#76	ERR715876	2010904	2842	ERP004245
ERS514532	14355_5#77	ERR715877	2010939	2842	ERP004245
ERS514534	14355_5#78	ERR715878	2010999	2842	ERP004245
ERS514536	14355_5#79	ERR715879	2011023	2842	ERP004245
ERS514537	14355_5#80	ERR715880	2011029	2842	ERP004245
ERS514539	14355_5#81	ERR715881	2011043	2842	ERP004245
ERS514542	14355_5#82	ERR715882	2011060	2842	ERP004245
ERS514543	14355_5#83	ERR715883	2011098	2842	ERP004245
ERS514545	14355_5#84	ERR715884	2011148	2842	ERP004245
ERS514547	14355_5#85	ERR715885	2011150	2842	ERP004245
ERS514549	14355_5#86	ERR715886	2011212	2842	ERP004245
ERS514551	14355_5#87	ERR715887	2011215	2842	ERP004245
ERS514553	14355_5#88	ERR715888	2011233	2842	ERP004245
ERS514555	14355_5#89	ERR715889	2011246	2842	ERP004245
ERS514557	14355_5#90	ERR715890	2011332	2842	ERP004245
ERS514559	14355_5#91	ERR715891	2011334	2842	ERP004245
ERS514560	14355_5#92	ERR715892	2011337	2842	ERP004245
ERS514563	14355_5#93	ERR715893	2011528	2842	ERP004245
ERS514565	14355_5#94	ERR715894	2011564	2842	ERP004245
ERS514566	14355_5#95	ERR715895	2011595	2842	ERP004245
ERS514286	14355_6#1	ERR715896	2011728	2842	ERP004245
ERS514289	14355_6#2	ERR715897	2011745	2842	ERP004245
ERS514292	14355_6#3	ERR715898	2011764	2842	ERP004245
ERS514293	14355_6#4	ERR715899	2011814	2842	ERP004245
ERS514296	14355_6#5	ERR715900	2011831	2842	ERP004245
ERS514299	14355_6#6	ERR715901	2011832	2842	ERP004245

ERS514301	14355_6#7	ERR715902	2011833	2842	ERP004245
ERS514303	14355_6#8	ERR715903	2011851	2842	ERP004245
ERS514305	14355_6#9	ERR715904	2011973	2842	ERP004245
ERS514308	14355_6#10	ERR715905	2011979	2842	ERP004245
ERS514311	14355_6#11	ERR715906	2012202	2842	ERP004245
ERS514314	14355_6#12	ERR715907	2012239	2842	ERP004245
ERS514317	14355_6#13	ERR715908	2012278	2842	ERP004245
ERS514319	14355_6#14	ERR715909	2012280	2842	ERP004245
ERS514322	14355_6#15	ERR715910	2012303	2842	ERP004245
ERS514324	14355_6#16	ERR715911	2012326	2842	ERP004245
ERS514327	14355_6#17	ERR715912	2012431	2842	ERP004245
ERS514330	14355_6#18	ERR715913	2012552	2842	ERP004245
ERS514332	14355_6#19	ERR715914	2012598	2842	ERP004245
ERS514334	14355_6#20	ERR715915	2012602	2842	ERP004245
ERS514339	14355_6#21	ERR715916	2012620	2842	ERP004245
ERS514340	14355_6#22	ERR715917	2012640	2842	ERP004245
ERS514343	14355_6#23	ERR715918	2012655	2842	ERP004245
ERS514346	14355_6#24	ERR715919	2012673	2842	ERP004245
ERS514349	14355_6#25	ERR715920	2020047	2842	ERP004245
ERS514355	14355_6#27	ERR715921	2020149	2842	ERP004245
ERS514358	14355_6#28	ERR715922	2020150	2842	ERP004245
ERS514359	14355_6#29	ERR715923	2020151	2842	ERP004245
ERS514362	14355_6#30	ERR715924	2020165	2842	ERP004245
ERS514365	14355_6#31	ERR715925	2020193	2842	ERP004245
ERS514368	14355_6#32	ERR715926	2020207	2842	ERP004245
ERS514370	14355_6#33	ERR715927	2020226	2842	ERP004245
ERS514372	14355_6#34	ERR715928	2020276	2842	ERP004245
ERS514375	14355_6#35	ERR715929	2020324	2842	ERP004245
ERS514378	14355_6#36	ERR715930	2020328	2842	ERP004245
ERS514380	14355_6#37	ERR715931	2020383	2842	ERP004245
ERS514382	14355_6#38	ERR715932	2020416	2842	ERP004245
ERS514385	14355_6#39	ERR715933	2020417	2842	ERP004245
ERS514388	14355_6#40	ERR715934	2020434	2842	ERP004245
ERS514390	14355_6#41	ERR715935	2020435	2842	ERP004245
ERS514392	14355_6#42	ERR715936	2020449	2842	ERP004245
ERS514395	14355_6#43	ERR715937	2020473	2842	ERP004245
ERS514398	14355_6#44	ERR715938	2020479	2842	ERP004245
ERS514401	14355_6#45	ERR715939	2020503	2842	ERP004245
ERS514402	14355_6#46	ERR715940	2020546	2842	ERP004245
ERS514405	14355_6#47	ERR715941	2020547	2842	ERP004245
ERS514408	14355_6#48	ERR715942	2020561	2842	ERP004245
ERS514411	14355_6#49	ERR715943	2020622	2842	ERP004245
ERS514414	14355_6#50	ERR715944	2020707	2842	ERP004245
ERS514417	14355_6#51	ERR715945	2020745	2842	ERP004245
ERS514421	14355_6#52	ERR715946	2020786	2842	ERP004245
ERS514423	14355_6#53	ERR715947	2020798	2842	ERP004245

ERS514426	14355_6#54	ERR715948	2020799	2842	ERP004245
ERS514427	14355_6#55	ERR715949	2020843	2842	ERP004245
ERS514430	14355_6#56	ERR715950	2060092	2842	ERP004245
ERS514432	14355_6#57	ERR715951	2060516	2842	ERP004245
ERS514434	14355_6#58	ERR715952	2060603	2842	ERP004245
ERS514437	14355_6#59	ERR715953	2060640	2842	ERP004245
ERS514439	14355_6#60	ERR715954	2060716	2842	ERP004245
ERS514442	14355_6#61	ERR715955	2060737	2842	ERP004245
ERS514444	14355_6#62	ERR715956	2060979	2842	ERP004245
ERS514446	14355_6#63	ERR715957	2061025	2842	ERP004245
ERS514449	14355_6#64	ERR715958	2061079	2842	ERP004245
ERS514450	14355_6#65	ERR715959	2061250	2842	ERP004245
ERS514453	14355_6#66	ERR715960	2061255	2842	ERP004245
ERS514457	14355_6#68	ERR715961	2061370	2842	ERP004245
ERS514460	14355_6#69	ERR715962	2061400	2842	ERP004245
ERS514463	14355_6#70	ERR715963	2061481	2842	ERP004245
ERS514465	14355_6#71	ERR715964	2061515	2842	ERP004245
ERS514467	14355_6#72	ERR715965	2061596	2842	ERP004245
ERS514470	14355_6#73	ERR715966	2061655	2842	ERP004245
ERS514473	14355_6#74	ERR715967	2070069	2842	ERP004245
ERS514475	14355_6#75	ERR715968	2070080	2842	ERP004245
ERS514477	14355_6#76	ERR715969	2070093	2842	ERP004245
ERS514479	14355_6#77	ERR715970	2070150	2842	ERP004245
ERS514481	14355_6#78	ERR715971	2070151	2842	ERP004245
ERS514484	14355_6#79	ERR715972	2070189	2842	ERP004245
ERS514487	14355_6#80	ERR715973	2070215	2842	ERP004245
ERS514489	14355_6#81	ERR715974	2070308	2842	ERP004245
ERS514491	14355_6#82	ERR715975	2070339	2842	ERP004245
ERS514494	14355_6#83	ERR715976	2070395	2842	ERP004245
ERS514497	14355_6#84	ERR715977	2070406	2842	ERP004245
ERS514500	14355_6#85	ERR715978	2070506	2842	ERP004245
ERS514502	14355_6#86	ERR715979	2070591	2842	ERP004245
ERS514504	14355_6#87	ERR715980	2070737	2842	ERP004245
ERS514507	14355_6#88	ERR715981	2070780	2842	ERP004245
ERS514510	14355_6#89	ERR715982	2070862	2842	ERP004245
ERS514513	14355_6#90	ERR715983	2070929	2842	ERP004245
ERS514516	14355_6#91	ERR715984	2071065	2842	ERP004245
ERS514519	14355_6#92	ERR715985	2071109	2842	ERP004245
ERS514521	14355_6#93	ERR715986	2071236	2842	ERP004245
ERS514524	14355_6#94	ERR715987	2071276	2842	ERP004245
ERS514527	14355_6#95	ERR715988	2071282	2842	ERP004245
ERS514950	14355_7#3	ERR715991	920550 I	2842	ERP004245
ERS514955	14355_7#6	ERR715994	920618 I	2842	ERP004245
ERS514959	14355_7#8	ERR715996	920619 I	2842	ERP004245
ERS514965	14355_7#11	ERR715999	920858 I	2842	ERP004245
ERS514970	14355_7#13	ERR716001	921206 I	2842	ERP004245

ERS514973	14355_7#15	ERR716003	921940 I		2842	ERP004245
ERS514980	14355_7#18	ERR716006	922049 I		2842	ERP004245
ERS514983	14355_7#20	ERR716008	922173 I		2842	ERP004245
ERS514987	14355_7#22	ERR716010	930164 I		2842	ERP004245
ERS514993	14355_7#25	ERR716013	930470 I		2842	ERP004245
ERS515001	14355_7#29	ERR716017	930524 I		2842	ERP004245
ERS515005	14355_7#31	ERR716019	930633 I		2842	ERP004245
ERS515013	14355_7#35	ERR716023	931069 I		2842	ERP004245
ERS515019	14355_7#38	ERR716026	940573 I		2842	ERP004245
ERS515021	14355_7#40	ERR716028	940605 I A		2842	ERP004245
ERS515022	14355_7#41	ERR716029	940605 I B		2842	ERP004245
ERS515024	14355_7#43	ERR716031	940655 I		2842	ERP004245
ERS515026	14355_7#45	ERR716033	940895 I		2842	ERP004245
ERS515028	14355_7#47	ERR716035	940908 I		2842	ERP004245
ERS515031	14355_7#50	ERR716038	941761 I		2842	ERP004245
ERS515034	14355_7#53	ERR716041	950733 I		2842	ERP004245
ERS515037	14355_7#56	ERR716044	951018 I		2842	ERP004245
ERS515039	14355_7#58	ERR716046	951249 I		2842	ERP004245
ERS515042	14355_7#61	ERR716049	951529 I		2842	ERP004245
ERS515044	14355_7#63	ERR716051	960444 I		2842	ERP004245
ERS515047	14355_7#66	ERR716054	961265 I		2842	ERP004245
ERS515049	14355_7#68	ERR716056	962030 I		2842	ERP004245
ERS515051	14355_7#70	ERR716058	970208 I		2842	ERP004245
ERS515053	14355_7#72	ERR716060	970710 I		2842	ERP004245
ERS515055	14355_7#74	ERR716062	971589 I		2842	ERP004245
ERS515058	14355_7#77	ERR716065	971612 I		2842	ERP004245
ERS515060	14355_7#79	ERR716067	971859 I		2842	ERP004245
ERS515062	14355_7#81	ERR716069	971982 I		2842	ERP004245
ERS515064	14355_7#83	ERR716071	980082 I		2842	ERP004245
ERS515066	14355_7#85	ERR716073	980748 I		2842	ERP004245
ERS515069	14355_7#88	ERR716076	980792 I		2842	ERP004245
ERS515072	14355_7#91	ERR716079	981287 I		2842	ERP004245
ERS515075	14355_7#94	ERR716082	981338 I		2842	ERP004245
ERS514954	14355_8#2	ERR716085	981564 I		2842	ERP004245
ERS514960	14355_8#5	ERR716088	982330 I		2842	ERP004245
ERS514966	14355_8#8	ERR716091	990447 I		2842	ERP004245
ERS514972	14355_8#11	ERR716094	990612 I		2842	ERP004245
ERS514977	14355_8#14	ERR716097	991441 I		2842	ERP004245
ERS514981	14355_8#16	ERR716099	991933 I		2842	ERP004245
ERS514988	14355_8#19	ERR716102	2000202 I		2842	ERP004245
ERS514994	14355_8#22	ERR716105	2010815 I		2842	ERP004245
ERS514997	14355_8#24	ERR716107	2010934 I		2842	ERP004245
ERS515002	14355_8#26	ERR716109	2011245 I		2842	ERP004245
ERS515008	14355_8#29	ERR716112	2012554 I		2842	ERP004245
ERS515012	14355_8#31	ERR716114	2030722 I		2842	ERP004245
ERS515016	14355_8#33	ERR716116	2031439 I		2842	ERP004245

ERS514770	14555_6#51	ERR731263	970636	2842	ERP004245
ERS514772	14555_6#52	ERR731264	970701	2842	ERP004245
ERS514774	14555_6#53	ERR731265	970974	2842	ERP004245
ERS514776	14555_6#54	ERR731266	971285	2842	ERP004245
ERS514778	14555_6#55	ERR731267	972054	2842	ERP004245
ERS514781	14555_6#57	ERR731269	980025	2842	ERP004245
ERS514784	14555_6#58	ERR731270	981146	2842	ERP004245
ERS514788	14555_6#60	ERR731272	990540	2842	ERP004245
ERS514792	14555_6#62	ERR731274	991076	2842	ERP004245
ERS514794	14555_6#63	ERR731275	2001532	2842	ERP004245
ERS514796	14555_6#64	ERR731276	780733 I	2842	ERP004245
ERS514800	14555_6#66	ERR731278	790143 I	2842	ERP004245
ERS514804	14555_6#68	ERR731280	790200 I	2842	ERP004245
ERS514810	14555_6#71	ERR731283	790555 I	2842	ERP004245
ERS514816	14555_6#74	ERR731286	800879 I	2842	ERP004245
ERS514820	14555_6#76	ERR731288	811198 I	2842	ERP004245
ERS514826	14555_6#79	ERR731291	820287 I	2842	ERP004245
ERS514830	14555_6#81	ERR731293	830088 I	2842	ERP004245
ERS514835	14555_6#84	ERR731296	840395 I	2842	ERP004245
ERS514839	14555_6#86	ERR731298	841159 I	2842	ERP004245
ERS514843	14555_6#88	ERR731300	841367 I	2842	ERP004245
ERS514848	14555_6#91	ERR731303	850782 I	2842	ERP004245
ERS514850	14555_6#93	ERR731305	850955 I	2842	ERP004245
ERS514283	14672_1#1	ERR743244	981781	2842	ERP004245
ERS514285	14672_1#2	ERR743245	981784	2842	ERP004245
ERS514288	14672_1#3	ERR743246	981826	2842	ERP004245
ERS514291	14672_1#4	ERR743247	981827	2842	ERP004245
ERS514295	14672_1#5	ERR743248	981871	2842	ERP004245
ERS514298	14672_1#6	ERR743249	981915	2842	ERP004245
ERS514302	14672_1#7	ERR743250	981936	2842	ERP004245
ERS514306	14672_1#8	ERR743251	982029	2842	ERP004245
ERS514309	14672_1#9	ERR743252	982102	2842	ERP004245
ERS514312	14672_1#10	ERR743253	982144	2842	ERP004245
ERS514315	14672_1#11	ERR743254	982153	2842	ERP004245
ERS514320	14672_1#12	ERR743255	982199	2842	ERP004245
ERS514323	14672_1#13	ERR743256	982200	2842	ERP004245
ERS514326	14672_1#14	ERR743257	982245	2842	ERP004245
ERS514329	14672_1#15	ERR743258	982340	2842	ERP004245
ERS514333	14672_1#16	ERR743259	982347	2842	ERP004245
ERS514335	14672_1#17	ERR743260	982375	2842	ERP004245
ERS514337	14672_1#18	ERR743261	990001	2842	ERP004245
ERS514342	14672_1#19	ERR743262	990005	2842	ERP004245
ERS514344	14672_1#20	ERR743263	990030	2842	ERP004245
ERS514347	14672_1#21	ERR743264	990056	2842	ERP004245
ERS514350	14672_1#22	ERR743265	990062	2842	ERP004245
ERS514353	14672_1#23	ERR743266	990069	2842	ERP004245

ERS514356	14672_1#24	ERR743267	990082	2842	ERP004245
ERS514361	14672_1#25	ERR743268	990092	2842	ERP004245
ERS514363	14672_1#26	ERR743269	990104	2842	ERP004245
ERS514366	14672_1#27	ERR743270	990121	2842	ERP004245
ERS514369	14672_1#28	ERR743271	990134	2842	ERP004245
ERS514373	14672_1#29	ERR743272	990135	2842	ERP004245
ERS514376	14672_1#30	ERR743273	990149	2842	ERP004245
ERS514379	14672_1#31	ERR743274	990163	2842	ERP004245
ERS514383	14672_1#32	ERR743275	990328	2842	ERP004245
ERS514386	14672_1#33	ERR743276	990334	2842	ERP004245
ERS514389	14672_1#34	ERR743277	990492	2842	ERP004245
ERS514393	14672_1#35	ERR743278	990502	2842	ERP004245
ERS514396	14672_1#36	ERR743279	990576	2842	ERP004245
ERS514399	14672_1#37	ERR743280	990601	2842	ERP004245
ERS514403	14672_1#38	ERR743281	990602	2842	ERP004245
ERS514406	14672_1#39	ERR743282	990615	2842	ERP004245
ERS514409	14672_1#40	ERR743283	990653	2842	ERP004245
ERS514412	14672_1#41	ERR743284	990738	2842	ERP004245
ERS514415	14672_1#42	ERR743285	990797	2842	ERP004245
ERS514418	14672_1#43	ERR743286	990808	2842	ERP004245
ERS514420	14672_1#44	ERR743287	990815	2842	ERP004245
ERS514424	14672_1#45	ERR743288	990907	2842	ERP004245
ERS514428	14672_1#46	ERR743289	990947	2842	ERP004245
ERS514431	14672_1#47	ERR743290	990975	2842	ERP004245
ERS514435	14672_1#48	ERR743291	991027	2842	ERP004245
ERS514441	14672_1#50	ERR743292	991056	2842	ERP004245
ERS514445	14672_1#51	ERR743293	991093	2842	ERP004245
ERS514448	14672_1#52	ERR743294	991097	2842	ERP004245
ERS514451	14672_1#53	ERR743295	991117	2842	ERP004245
ERS514454	14672_1#54	ERR743296	991159	2842	ERP004245
ERS514458	14672_1#55	ERR743297	991174	2842	ERP004245
ERS514462	14672_1#56	ERR743298	991192	2842	ERP004245
ERS514466	14672_1#57	ERR743299	991208	2842	ERP004245
ERS514469	14672_1#58	ERR743300	991210	2842	ERP004245
ERS514471	14672_1#59	ERR743301	991247	2842	ERP004245
ERS514474	14672_1#60	ERR743302	991275	2842	ERP004245
ERS514478	14672_1#61	ERR743303	991344	2842	ERP004245
ERS514482	14672_1#62	ERR743304	991379	2842	ERP004245
ERS514485	14672_1#63	ERR743305	991382	2842	ERP004245
ERS514488	14672_1#64	ERR743306	991397	2842	ERP004245
ERS514492	14672_1#65	ERR743307	991511	2842	ERP004245
ERS514496	14672_1#66	ERR743308	991598	2842	ERP004245
ERS514499	14672_1#67	ERR743309	991625	2842	ERP004245
ERS514501	14672_1#68	ERR743310	991642	2842	ERP004245
ERS514505	14672_1#69	ERR743311	991661	2842	ERP004245
ERS514511	14672_1#71	ERR743313	991712	2842	ERP004245

ERS514514	14672_1#72	ERR743314	991722	2842	ERP004245
ERS514520	14672_1#74	ERR743315	991774	2842	ERP004245
ERS514523	14672_1#75	ERR743316	991853	2842	ERP004245
ERS514526	14672_1#76	ERR743317	991930	2842	ERP004245
ERS514529	14672_1#77	ERR743318	991932	2842	ERP004245
ERS514531	14672_1#78	ERR743319	992008	2842	ERP004245
ERS514533	14672_1#79	ERR743320	992062	2842	ERP004245
ERS514535	14672_1#80	ERR743321	992073	2842	ERP004245
ERS514538	14672_1#81	ERR743322	992076	2842	ERP004245
ERS514540	14672_1#82	ERR743323	2000020	2842	ERP004245
ERS514541	14672_1#83	ERR743324	2000024	2842	ERP004245
ERS514544	14672_1#84	ERR743325	2000041	2842	ERP004245
ERS514546	14672_1#85	ERR743326	2000070	2842	ERP004245
ERS514548	14672_1#86	ERR743327	2000100	2842	ERP004245
ERS514550	14672_1#87	ERR743328	2000101	2842	ERP004245
ERS514552	14672_1#88	ERR743329	2000131	2842	ERP004245
ERS514554	14672_1#89	ERR743330	2000136	2842	ERP004245
ERS514556	14672_1#90	ERR743331	2000149	2842	ERP004245
ERS514558	14672_1#91	ERR743332	2000151	2842	ERP004245
ERS514561	14672_1#92	ERR743333	2000194	2842	ERP004245
ERS514562	14672_1#93	ERR743334	2000201	2842	ERP004245
ERS514564	14672_1#94	ERR743335	2000234	2842	ERP004245
ERS514567	14672_1#95	ERR743336	2000236	2842	ERP004245
ERS514438	14893_3#67	ERR775252	991044	2842	ERP004245
ERS514517	14893_3#68	ERR775253	991765	2842	ERP004245
ERS514352	14893_3#69	ERR775254	2020094	2842	ERP004245
ERS514456	14893_3#70	ERR775255	2061285	2842	ERP004245
