# Supplementary material for: Genetic Variation in Neisseria meningitidis Does Not Influence Disease Severity in Meningococcal Meningitis
Source: Front Med (Lausanne). 2020 Nov 11;7:594769. doi: 10.3389/fmed.2020.594769 (PMC7686797; doi:10.3389/fmed.2020.594769)
Supplement: Supplementary file 3 [file Table_2.pdf]

Supplementary Table S2

CC	Favorable		Unfavorable	
	Number	%	Number	%
ST-41/44	128	90	14	10
ST-32	57	86	9	14
ST-11	56	81	13	19
ST-269	24	92	2	8
ST-213	11	92	1	8
Other	49	91	5	9
Serogroup	Number	%	Number	%
B	237	90	27	10
C	70	82	15	18
W135	5	100	0	0
Y	12	92	1	8
X	1	100	0	0
A	0	0	0	0
29E	0	0	1	100
